# Mechanical Studies of the Third Dimension in Cancer: From 2D to 3D Model

**DOI:** 10.3390/ijms221810098

**Published:** 2021-09-18

**Authors:** Francesca Paradiso, Stefano Serpelloni, Lewis W. Francis, Francesca Taraballi

**Affiliations:** 1 Center for Musculoskeletal Regeneration, Houston Methodist Research Institute, 6670 Bertner Ave, Houston, TX 77030, USA; fparadiso@houstonmethodist.org (F.P.); sserpelloni@houstonmethodist.org (S.S.); 2 Orthopedics and Sports Medicine, Houston Methodist Hospital, 6445 Main St., Houston, TX 77030, USA; 3 Reproductive Biology and Gynaecological Oncology Group, Swansea University Medical School, Singleton Park, Swansea, Wales SA2 8PP, UK; l.francis@swansea.ac.uk

**Keywords:** 3D model, cancer, microenvironment, biomaterials, mechanics, mechanosensing

## Abstract

From the development of self-aggregating, scaffold-free multicellular spheroids to the inclusion of scaffold systems, 3D models have progressively increased in complexity to better mimic native tissues. The inclusion of a third dimension in cancer models allows researchers to zoom out from a significant but limited cancer cell research approach to a wider investigation of the tumor microenvironment. This model can include multiple cell types and many elements from the extracellular matrix (ECM), which provides mechanical support for the tissue, mediates cell-microenvironment interactions, and plays a key role in cancer cell invasion. Both biochemical and biophysical signals from the extracellular space strongly influence cell fate, the epigenetic landscape, and gene expression. Specifically, a detailed mechanistic understanding of tumor cell-ECM interactions, especially during cancer invasion, is lacking. In this review, we focus on the latest achievements in the study of ECM biomechanics and mechanosensing in cancer on 3D scaffold-based and scaffold-free models, focusing on each platform’s level of complexity, up-to-date mechanical tests performed, limitations, and potential for further improvements.

## 1. Introduction

### 1.1. Mechanosensing: A Biophysical Signal Travels from the Outside to the Inside of the Cells

**Figure 1:**
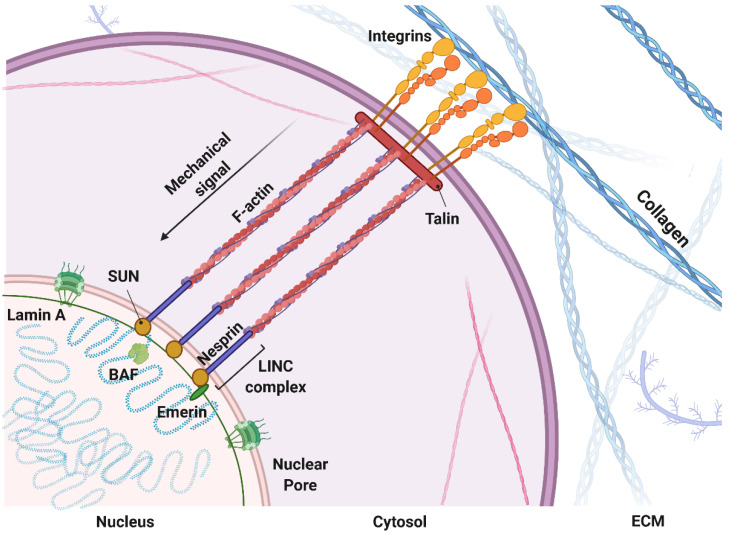
Mechanical coupling of the extracellular matrix with the nucleus: Mechanical signal propagation through molecular pathways inside the cytosol and nucleus. Made with Biorender.

Today, tumors are depicted as organs, with a higher complexity compared to normal healthy tissue [1]. The complexity of a neoplastic disease relies on individual specialized cells within the tumor tissue that sustain the ability of cancer cells to build a flourishing niche in order to grow and spread, called the tumor microenvironment (TME) [2]. Tumor cells and mesenchymal cells, forming the tumor-associated stroma, collaborate in a multistep process to create the overall tumor tissue (composed of cells and extracellular matrix [ECM]), which is very different from its healthy counterpart in biological, mechanical, chemical, and topographic characteristics [3,4]. During tumor development, cancer cells and stromal cells remodel the surrounding microenvironment and consequently affect and are affected by both biochemical and biophysical signals derived from it [5,6]. For example, the biophysical signal derived from changes in ECM stiffness is a solid tumor-specific trigger that enhances epithelial cells to transition into a malignant phenotype [7]. Alterations in tumor mechanics can derive from imbalances between matrix deposition and degradation [8,9,10]; increases in matrix crosslinking [11,12]; defective lymphatic drainage and leaking from blood vessels (increase in oncotic and fluid pressure) [13,14]; and deregulated growth and high cell densities that generate solid pressure and cell “jamming”, which prevents cell movements [15,16]. These mechanical signals are transduced into biochemical signals from the cytoskeleton to the nucleus, actively affecting tumor cell behavior (Figure 1). Biophysical signals are mostly conveyed by membrane-spanning dimers called integrin receptors, which bind to the surrounding ECM and physically bridge them to the cell cytoskeleton, translating mechanical signals into biochemical ones [17,18,19]. In this way, focal adhesions and integrin receptors serve as biochemical signaling hubs to initiate mechanoresponsive signaling pathways by concentrating and directing signaling proteins involved in cell polarity, response to tensile strength, migration, and invasion [20,21]. Mechanical signals transduced from the external microenvironment to the intracellular cytoskeleton can be further transmitted into the nucleus, where they can affect nuclear architecture and chromosome and chromatin organization, resulting in both genetic and epigenetic landscape changes [22]. Analogous to focal adhesions, linkers of nucleoskeleton and cytoskeleton protein (LINC) complexes on the nuclear membrane physically connect the cytoskeleton to the nucleoskeleton [23,24]. This complex comprises two families: the KASH domain proteins (nesprins) bind to various cytoskeletal constituents, whereas the SUN domain proteins associate with the nuclear lamina (laminins) and nuclear pore complexes (NPCs) [25]. Laminins connect directly and indirectly through emerin and lamin B receptor (LBR) binding to different regulatory proteins that are involved in chromatin modification, transcriptional regulation, and mRNA processing, and to BAF protein (or BANF1), which binds directly to double-stranded DNA [26]. These anchor complexes that connect the ECM to the nucleus through the cytoskeleton can alter the DNA regulatory complex activity with consequent changes in chromatin organization [27], gene transcription [28], RNA splicing, or chromatin modification [26,29].

### 1.2. Mechanomodeling: Inclusion of Biophysical Signals in 3D Cancer Systems

Dissection of the effect of biophysical signals on cancer cells during tumor development has become the focus of many in vitro studies. The simplicity, low cost, and reproducibility of 2D models made them the mainstay of biological research, but in vivo tissue complexity can only be approached using 3D systems. In fact, in tridimensional systems, the mechanical properties can be tuned so that models mimic a wide range of tissue stiffness [30,31,32]. Moreover, cell adhesion, spreading, and migration is not constrained to a single layer [33,34,35]; sequestration/gradients of soluble biomolecules can be modulated to finely control cell fate and differentiation [36,37]; and ECM can be customized to reproduce the in vivo cell experience through different sets of chemical and mechanical signals [38,39,40]. Many processes are intrinsically tied to cell–cell and cell–matrix interactions, whether through synthesis, degradation, directed migration, or mechanical cues, and cannot be fully reproduced in conventional 2D cell culture [41,42,43,44], e.g., cancer metastasis or cancer–stroma interaction [45,46,47,48].

**Figure 2:**
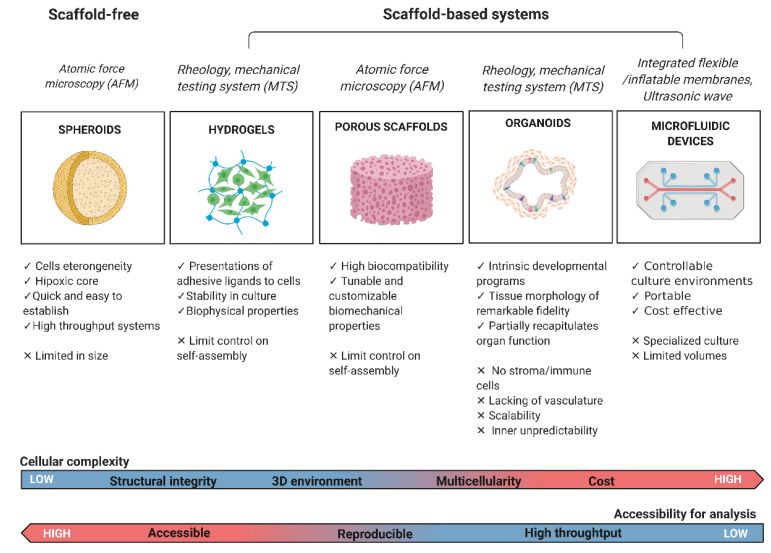
Classification of the most common cancer study models with their strengths and limitations. The primary mechanical tests performed on each platform are reported in italics. Biological and technical characteristics of the cancer model are highlighted. On the bottom, 3D in vitro systems cellular complexity and accessibility of imaging and analysis. Made with Biorender.

The inclusion of mechanical constraints in designing an in vitro model requires the use of 3D culture platforms (scaffold-free or scaffold-based approaches) to fully mimic native tumor tissue biology as well as mechanical and biochemical properties [49]. The use of scaffold-based approaches to growing cells in a 3D environment is very common in tissue engineering [50]. The challenges of reproducing microenvironment features in a 3D model fueled the scientific community to develop a wide variety of platforms to address different levels of complexity, e.g., cells can be seeded on pre-formed porous scaffolds/fibrous materials (obtained by two-phase emulsion, freeze-drying, or electro-spinning techniques) or encapsulated in biomaterials made of water-soluble polymers called hydrogels [51,52,53]. Another promising trend is the use of native ECM obtained by tissue decellularization, employed as a scaffold for cell seeding or as an additive component of 3D gels in order to mimic in vitro the ECM architecture and chemical/biological properties [54,55,56]. Furthermore, tissue physiology can be reproduced with the use of adult or pluripotent stem cell-derived organoids, which are self-organized 3D tissue cultures crafted from stem cells to replicate part or much of the complexity of an organ; alternatively, cells can be grown in a multi-channel 3D microfluidic cell culture chip that simulates mechanics, activities, and physiological response of specific organs or systems. All these platforms have different levels of complexity and can reproduce certain mechanical features from the native tissue that will be discussed in the following sections (Figure 2).

### 1.3. Mechanotesting: Technologies to Approach Biophysical Studies in 3D Cancer Modeling

The studies of mechanics in 3D systems for cancer research have enabled researchers to develop better technologies and adapt protocols from material science to cancer biology. So far, at a cellular level, mechanics can be measured with different methods probing stiffness at the nano-scale and micro-scale, including micropipette aspiration [57,58,59] and optical stretcher [60] for measuring mechanics of whole cells in suspensions, and atomic force microscopy (AFM) for the investigation of single adherent cells [61,62,63,64]. For example, many studies used AFM on different types of epithelial cancer cells, showing that cancer cells are generally softer and display lower intrinsic variability in cell stiffness than non-malignant cells [65,66]. Furthermore, AFM was successfully applied to measure the mechanics of tumor spheroids to promote understanding of tumor growth in confined environments, showing that tumor spheroids grown in stiff hydrogels were significantly stiffer than those grown in compliant hydrogels [67]. In addition, AFM can reveal the mechanical dynamics of the basement membrane during the invasion process of tumor cells. Morphological imaging by AFM showed that the basement membrane cultured with cancer and stromal cells had higher roughness and more holes during the tumor breaching process but became softer upon cancer cell and fibroblast growth, clearly suggesting basement membrane mechanics are dynamic during cancer invasion and metastasis [68].

Elastic and viscoelastic properties of tissues, cells, and ECM are typically measured using rheology, the study of flow and deformation of matter, to characterize both the elastic (Gʹ, storage modulus) and viscous (Gʹʹ, loss modulus) behavior. Rheology measurements provide an interesting tool to study the interaction between forces and the flow/deformation of materials that exhibit a combination of elastic, viscous, and plastic behavior, like normal and tumor tissues [69,70]. Tissues are composed of colloidal particles, filamentous polymers, and other supra-molecular arrangements, leading to complicated deformations in response to mechanical stress. Rheological measurement probes the mechanical responses of viscoelastic media but also establishes predictions for mechanical behavior based on the micro- or nanostructure of the material [71]. Finally, bulk compression or tension analysis is used to measure the elastic modulus (E; Young’s modulus), which relates to the architecture of the bulk tissue or the 3D model under investigation [72,73,74].

The overall mechanical properties of a tissue are meaningful in assessing its physical characteristics during processes such as cancer development [75]. Although the TME is of critical importance during the initiation and spread of cancer, relatively little is known about its biophysical evolution and how it impacts nuclear processes inside the cells, such as epigenetic regulation, which have direct consequences on gene expression. Unfortunately, there are few reports addressing the link between tissue mechanics and epigenetics in 3D systems, but we hope that this link will be filled by the growing interest in the field and technological advances. In this review, we explore the latest achievements in the field of 3D culturing systems used for the dissection of solid tumor TME mechanical changes and the reported effects on cancer and stromal cell gene expression, behavior, and epigenetic machinery.

## 2. Scaffold-Free 3D Models

### 2.1. Tumour Spheroids

Spheroids are 3D aggregates of a single cell type or heterogeneous cell populations [76]. Cell–cell interactions orchestrate aggregate formation without the support of a scaffolding material while producing their own ECM. Four major techniques are used to induce tumor spheroid formation in vitro: agitation-based techniques, liquid overlay techniques, hanging-drop techniques, and microfluidic reactors [77,78]. These cultures allow for the recapitulation of important features of TME heterogeneity, such as oxygen gradients and immune infiltration, and they are relatively quick and easy to establish, representing a useful tool for high throughput systems [79]. However, they can be limited in size by the lack of nutrition diffusion to cells inside the cultures, depending on the size and the self-assembling process of cells [80,81]. Overall, the transport of nutrients to the cells within a tissue in the body is facilitated by either convection of blood in the vascular system and convection within the interstitial fluid. In avascular tissues, including many of the 3D in vitro models developed, nutrient delivery relies on passive diffusion [82]. Passive diffusion of either gas or nutrients in engineered constructs is an important design parameter [83] that can impact cell viability, differentiation, or function, i.e., lack of oxygen (hypoxia) [84,85]. However, oxygen gradients and hypoxia areas can be successfully used to investigate tumor cell adaptations under hypoxia, mimicking avascular tumor spaces, since both play key roles in tumor progression and resistance to treatment [86,87]. For example, spheroids exceeding 400 μm in diameter develop a hypoxic core and activate known survival signaling pathways to maintain cell viability [88].

Spheroids can be obtained from immortal cell lines, for example, adenocarcinoma tumor cell lines (e.g., SKOV-3) and ovarian cancer metastatic cell lines (e.g., OVCAR-3) [89]. OVCAR-3 cells were recently used to create spheroids by forced floating in ultra-low attachment plates and hanging drop methods to perform drug response analysis with paclitaxel, a common drug used to treat ovarian cancer. Spheroids maintained the cell density and higher apoptosis behavior of ovarian cancer, as well as higher resistance against paclitaxel treatment when compared with 2D culture, closely mimicking the in vivo response [90]. Successful spheroids can also be generated from patient-derived tissue samples, such as 3D spheroid suspension cultures from radical prostatectomy specimens to model organ-confined prostate cancer. Cancerous tissue samples from radical prostatectomy specimens were excised by a uropathologist, and preparation of 3D spheroids was performed by mechanical disintegration and limited enzymatic digestion followed by serial filtrations. Spheroids formed successfully and remained viable for up to several months, serving as an innovative in vitro model of organ-confined prostate cancer [91]. The elective techniques used to detect the stiffness of single cells, including AFM or optical tweezers, can measure forces ranging from sub pN to a few hundred nN, which are not suitable for the measurement of larger 3D cellular structures such as spheroids, whose mechanical characteristics have not been fully studied. One strategy is to use microtweezers that measure forces from sub-hundred nN to mN. This wide force range was achieved by the use of a chopstick-like motion of two cantilevers, which facilitated easy handling of samples and microscopic observation for mechanical characterization of normal and cancerous spheroids [92].

Another interesting strategy to detect mechanical cues occurring during 3D cellular aggregate formation is the use of fabricated polyacrylamide microbeads as built-in pressure sensors to locally measure changes in mechanical stress in order to understand how a compressive stress (which occurs physiologically during tumor growth) is distributed within the 3D cellular aggregates. Dolega et al. reported that cells at the spheroid surface continuously proliferate and actively rearrange under compressive stress [67,93]. Hypothetically, the high availability of oxygen and nutrients at the surface leads the outer layer to proliferate, generating tension and contraction. Compressive stresses dissipate toward the less dense core of multi-cellular spheroids, where contractility is reduced as nutrients and oxygen are depleted. Others used similar microscopy-compatible probes that report quantitative, directional, and real-time measurements of stress at cellular- and supra-cellular length scales within engineered tissues to achieve localized measurements at the length scale of individual cells (10 s of μm) with a large dynamic range from 10–1000 s of Pa. Those studies found that cells located at the spheroid’s periphery exhibit greater contractility, as evidenced by increased expression of phosphorylated myosin, and that these differences in mechanical forces are associated with the activation of mechanosensitive signaling pathways involving YAP/TAZ transcription factors, which hypothetically could give rise to distinct differentiation profiles and spatially regulated biological activity [94].

Embedding multicellular spheroids within gels of defined mechanical properties (e.g., agarose gels) was one of the first experimental approaches to mimic tumor growth in a constraining environment, reproducing the compressive stress accumulation at the tumor-stroma interface [95]. In particular, the researchers reported that inhomogeneities in the mechanical properties of the confining tissue can guide morphological changes in tumor growth independent of cell migration by inducing apoptosis in regions of high compressive stress and allowing proliferation in regions of low stress [96]. These models can provide useful insight into how compression directly influences tumor development and disclose the role of mechanical stresses in the process.

For some tumors, such as epithelial ovarian cancer, the role of ECM stiffness and cellular mechanotransduction is still very understudied. Interestingly, it was reported that disaggregation of multicellular epithelial ovarian cancer spheroids, a behavior associated with dissemination and metastasis, is enhanced by matrix stiffness through a mechanotransduction pathway involving ROCK, actomyosin contractility, and FAK. This pattern of mechanosensitivity is maintained in highly metastatic SKOV3ip.1 cells [97]. As a result of microenvironment changes, epigenetic alterations can occur and play a key role in promoting transformation and tumor growth, although the underlying mechanisms are still being worked out. Analysis of spheroid versus monolayer ovarian cancer cells showed an increase of two epigenetic processes, DNA methylation and histone acetylation, specifically during 3D growth. These findings support the hypothesis that ovarian cells in three-dimensional (3D) culture are physiologically different from their monolayer counterparts. DNA methylation changes are seen in several cancer types and have been linked to changes in gene expression in highly metastatic tumors, while histone acetylation has important roles in diverse processes, including gene regulation, DNA damage repair, and DNA replication, although it has been associated with both better and worse prognoses depending on the specific cancer type [98].

To date, many kinds of spheroids have been developed, including neurospheres, mammospheres, hepatospheres, and embryoid bodies [99,100,101]. Spheroids can be easily analyzed by imaging using light fluorescence, and confocal microscopy and many other techniques are being optimized to gain knowledge on the mechanical features of those systems. The next step forward will be to link mechanical features of the ECM to mechanosensing pathways in the cells and potentially exploring the epigenetic landscape affected by those changes. Furthermore, while the generation of 3D cultures can be more labor-intensive than 2D culture, the routine incorporation of these multi-cellular 3D spheroids into in vitro drug efficacy and toxicity testing could effectively bridge the gap between in vitro 2D assessment and animal models of disease, fast-tracking drug screening and, hopefully, yielding more effective and less toxic drugs as future therapies [69].

### 2.2. Scaffold-Based 3D Models

#### 2.2.1. Hydrogels

The most represented materials in the regenerative medicine literature and pharmacological applications are polymeric hydrogels because of their multi-tunable properties and structural similarity to native ECM [102]. A hydrogel is a 3D network structure composed of cross-linked polymer chains, which has the ability to absorb a large volume of solution. Native ECM is a prototypical hydrogel that promotes cell viability and direct cell adhesion, differentiation, proliferation, and migration through the controlled presentation of mechanical and biochemical cues. Mechanical strength, nutrient transport, topography, and degradation behavior can be tuned by using polymers with different compositions, adjusting their crosslinking density, and inclusion of bioactive molecules (such as cell-adhesion ligands or proteolytic degradation sites) [103,104,105,106].

General advantages of hydrogel systems include the ability to present adhesive ligands to cells either in native or modified gel, stability of the cultured cells, and the option to modify biophysical properties (e.g., elastic modulus) [107].

Hydrogels for cell culture can be naturally derived, including protein-based (collagen, gelatin, silk fibroin, and elastin fibrin), polysaccharide-based (glycosaminoglycan, alginate, and chitosan), a combination of materials such as in Matrigel, or decellularized tissue-derived [108]. On the other hand, synthetic hydrogel materials comprise polyacrylamide, polyethylene glycol (PEG), polyvinyl alcohol, and poly-2-hydroxyethyl methacrylate and were first developed in the 1960s [109]. Naturally derived gels are biocompatible and bioactive but are hindered by inherent batch-to-batch variability and difficulties in tuning mechanics and biochemical properties, while synthetic hydrogels are highly reproducible and easy to manufacture, process, or tune for different mechanical studies [110,111]. They can be degraded by cells and allow for ECM deposition, but as inert materials, they lack some of the native ECM complexity and biological signals, which therefore must be implemented in those models [112].

Assessment of the mechanical properties of a hydrogel system is usually performed at the macro scale (using tensional or shear rheometry) or using high-resolution tools, such as AFM, which are suited for sub-cellular nanoscale measurements and are limited to measuring near-surface stiffness in 2D or cut tissue sections [113]. Other non-contact techniques such as ultrasound elastography or magnetic cytometry can give information at the macro scale but cannot capture local mechanical variations around cells or mimic a cell’s ability to interrogate its surroundings [114]. These technologies can support impactful research but also limit our technical ability to monitor and characterize tissue mechanics at the cellular length scale during tissue morphogenesis and disease.

A wide range of studies have developed hydrogels consisting of interpenetrating networks (IPNs) of reconstituted basal membrane (rBM) to offer a platform with ligands and nanoporosity similar to native BM, stiffness resembling that of the tumor tissue, and limited susceptibility to cell-mediated degradation. For example, IPNs of rBM matrix and alginate can be used to modulate ECM stiffness and explore the effect of stiffness vs. BM ligand display in normal and malignant mammary epithelial cells. The mechanical properties of the IPNs and pure rBM matrix were characterized with an AR-G2 stress-controlled rheometer (TA Instruments). This composite alginate–Matrigel hydrogel used a controlled degree of ionic cross-linking of alginate with Ca^2+^ ions, allowing the matrix stiffness to be altered independently of ligand density and architecture for 3D cell culture. It revealed that, in normal mammary epithelial cells, increasing ECM stiffness alone induces malignant phenotypes, while increasing BM ligands completely abrogated this effect [7].

Another study reported a strategy to control the stiffness of a cell-encapsulating fibril collagen hydrogel while minimizing changes in gel permeability and the number of cell adhesion sites [115]. To demonstrate this strategy, cell-instructive hydrogels with controllable stiffness and limited changes in permeability were developed using interconnected collagen fibers with varied amounts of PEG di-(succinic acid N-hydroxysuccinimidyl ester). The compressive elastic moduli (E0) of the synthesized gels were measured by compressing the hydrogels with a mechanical testing system (MTS Insight). Growing hepatocellular carcinoma cells encapsulated in a fat-like soft PEG–cross-linked collagen hydrogel formed malignant spheroids, whereas cells cultured in a liver-like, stiffer gel formed spheroids with suppressed malignancy [115].

Another study from Wisdom et al. used IPNs of Matrigel and alginate to correlate hydrogel stiffness, recorded using an AR2000EX stress-controlled rheometer (TA Instruments), to human mammary tumor mechanics, recorded using an unconfined compression test with the Instron 5848 material testing system (1 N load cell; Futek). Exploiting this model, they reported a protease-independent migration in human breast adenocarcinoma, in which cells can migrate through confining matrix with sufficient mechanical plasticity, first utilizing invadopodia protrusions to mechanically and plastically open up channels and then generating protrusive forces at the leading edge to migrate through them [116].

Cell migration through ECM requires nuclear deformation, which is affected by nuclear stiffness. This feature is impacted by chromatin structure, specifically methyltransferase complexes, which comprise a catalytic subunit (SET/MLL) and several core scaffolding subunits, including WD repeat-containing protein 5 (WDR5), that can alter chromatin organization and thereby affect the physical properties of the nucleus. Wang et al. utilized AFM, reporting that the nuclei in 3D collagen type I-based matrix environments were softer and thereby more deformable compared with cells in suspension or cultured in 2D conditions. Furthermore, they determined that actomyosin contractility controls the interaction of WDR5 with other components of the methyltransferase complex through the phosphorylation of myosin by myosin light chain kinase (MLCK), which in turn up-regulates H3K4 methylation activation in 3D conditions. Through the methylation of histone H3 on residue K4 (H3K4me3), a hallmark for cancer progression and migration, WDR5 was reported to play a critical role in cell migration in vitro and in vivo [117].

Other hydrogel platforms were utilized to explore cancer cells’ responses to chemotherapeutic drugs, such as the use of a collagen–alginate hydrogel with a 20-fold variation in stiffness for the evaluation of estrogen receptor-positive breast cancer spheroid response to doxorubicin [118]. As in many other cases, the mechanical tuning was achieved by altering the degree of cross-linking of alginate molecules. As reported, soft hydrogels promote the growth of larger MCF-7 tumor spheroids with a lower fraction of proliferating cells and enhance spheroid resistance to doxorubicin [118]. Mechanical characteristics were assessed using a rheometer with a cone and plate geometry, performing a strain/frequency sweep and a time sweep to test the hydrogel’s viscoelastic response and finally obtaining the Young’s modulus of the hydrogel as E = 2G (1 + υ), where υ is the Poisson ratio and G is the shear modulus calculated as G = GI 2 + GII 2 (hydrogel storage and loss moduli).

Three-dimensional hydrogel scaffolds can also be designed as platforms for growing complex tissue structures. Mammary-like tissues from primary patient-derived cells were grown on engineered hydrogel scaffolds that incorporated both the protein (collagen, laminins, and fibronectin) and carbohydrate (hyaluronan) components of human breast tissue. Cells rapidly self-organized in the absence of stromal cells and expanded within 2 weeks to form mature tissues containing luminal, basal, and stem cells in the correct topological orientation and exhibited the complex ductal and lobular morphologies observed in the human breast [119]. A standard, widely used, 3D hydrogel system is Matrigel matrix. This platform is a BM extract (BME) consisting of collagen type IV, laminin, perlecan, etc. [120]. It represents an elective system to grow spheroid structures for many tumors, mimicking tissue morphology and unveiling new targets for therapy. For example, breast cancer cell lines grown on Matrigel showed that methylation of the TIMP3 promoter and changes in expression of TIMP3 and DNMT3B were all affected by miR-29c regulation. miR-29c was found to exert a suppressive role in the development of breast cancers, inhibiting cell migration and invasion. DNMT3B is mainly involved in de novo DNA methylation and its expression is inhibited by miR-29c targeting on the 3′UTR. Upregulation of DNMT3B is critical for promoter methylation and decreased expression of TIMP3, an inhibitor of extracellular matrix metalloproteinase that can suppress angiogenesis, tumor growth, invasion, and migration. In another study, a human normal colon epithelial cell line was grown in BME to form spheroids, which were then treated with conditioned media from a human colon myofibroblast line (CCD-18Co) to induce epithelial to mesenchymal transition in colon epithelial cells (HCoEpiCs). CCD-18Co-conditioned media triggered multiple morphological and epigenetic changes in HCoEpiCs, increased cell diameter, down-regulation of E-cadherin and up-regulation of vimentin and α-SMA, and up-regulation of HDAC1 and HDAC2. Epigenetic treatment with the HDAC inhibitor trichostatin A (TSA) reversed the CCD-18Co-induced morphological changes and migration of the HCoEpiCs and suppressed the downregulation of E-cadherin and upregulation of vimentin, α-SMA, ZEB1, and Snail, providing a useful therapeutic tool for the reversion of cancer-associated fibroblast (CAF)-induced epithelial to mesenchymal transition in colon epithelium [121].

Hydrogels can also be engineered to release therapeutics, modulating their retention time in the tissue. For example, Ruan et al. used an ROS-responsive hydrogel (Zeb-aPD1-NPs-Gel) cross-linked by mixing polyvinyl alcohol and N1-(4-boronobenzyl)-N3-(4-boronophenyl)-N1,N1,N3,N3-tetramethylpropane-1,3-diaminium (TSPBA) linker to utilize the acidic TME and ROS within tumors for the controlled release of zebularine, a demethylation agent, and aPD1 antibody. This combination therapy increased the immunogenicity of cancer cells, inhibiting the tumor growth and prolonging the survival time of B16F10-melanoma-bearing mice [122].

Crucial advances were obtained using thermoresponsive, smart material microgels, termed micro-scale temperature-actuated mechanosensors (μTAMs), which can be dispersed or injected into tissues and optically assayed to measure residual tissue elasticity after creep over several weeks. Using thermoresponsive hydrogels as smart 3D platforms, researchers reported focal sites of increased intratumoral rigidity in invasive breast cancer spheroids, and in an in vivo mouse model of breast cancer progression [123].

As discussed, the matrix stiffness not only affects migration properties, cellular response to therapeutics, and cell fate [124] but also the phenotype of cancer cells. Decoupling matrix stiffness cues from other characteristics in cancer studies can be achieved utilizing engineered hydrogels, but we need to overcome technical issues in conventional mechanical characterization techniques, particularly at the length scale of individual cells.

#### 2.2.2. Pre-Made Porous Scaffolds

One of the very first approaches employed in tissue engineering was seeding therapeutic cells in pre-made porous scaffolds of biodegradable materials, and over time, researchers developed many different types of biomaterials for multiple applications and different techniques, such as two-phase emulsions and foams, freeze-drying or electro-spinning, and two-photon lithography [125].

In general, there are two different sources for porous scaffold biomaterials, namely natural and synthetic. Natural biomaterials can derive from ECM allografts and xenografts, which can create potential immunogenicity, or they can be made of organic polymers, such as proteins, polysaccharides, lipids, and polynucleotides. Their high biocompatibility promotes cell adhesion and attachment for excellent cell proliferation and survival. Interestingly, new sources of natural collagen-like materials found in marine sources, including jellyfish R. pulmo collagen, are effective for the fabrication of 3D devices, such as sponges, to mimic tissue architecture complexity for cancer studies [126]. On the other hand, naturally derived scaffolds’ mechanical properties are poor, so they require the addition of synthetic materials and crosslinking for physical stabilization [127].

To this aim, synthetic biomaterials (poly(ε-caprolactone) [PCL], poly(glycolic acid) [PGA], poly(lactic acid) [PLA], and their derivatives) have been developed to overcome some natural scaffolds’ limitations, providing tunable and customizable biomechanical properties, compositions, and geometry to tailor both soft and hard materials [128]. Additional coatings are usually required on these materials to improve their biocompatibility.

The scaffold-based cultures provide a physiological context to cancer cells, addressing critical biological and mechanical cues needed to maintain morphological and genotypic tumorigenicity. For example, a 3D microscaffold was realized by two-photon lithography to explore the role of β-catenin in both mechanotransduction and tumorigenesis. This model showed that 3D microenvironments are sufficient per se to activate β-catenin-dependent mechanosensing circuits that can boost breast cancer cell proliferation and invasiveness. In particular, they observed that the polymeric 3D cage-like structures initiate nuclear relocation of β-catenin and vimentin expression in epithelial/non-invasive MCF-7 cells [129].

In another study, researchers showed that culturing breast cancer cells in 3D scaffolds that mimic the in vivo tumor-like microenvironment enhances their metastatic potential. They used porous PCL scaffolds of approximately modulus 7 kPa, comparable to that of breast tumor tissue, on which MDA-MB-231 cells proliferated and formed tumoroids. Gene expression analysis revealed that cells growing in the scaffolds expressed increased levels of genes implicated in the three major events of metastasis (initiation, progression, and site \-specific colonization), including cell-cell and cell-matrix interactions and tissue remodeling; cancer inflammation; and the PI3K/Akt, Wnt, NF-κB, and HIF1 signaling pathways, compared to cells grown in conventional 2D tissue culture polystyrene dishes. The cells cultured in scaffolds showed increased invasiveness and sphere formation efficiency in vitro and increased lung metastasis in vivo [130].

In line with the previous study, mixtures of rat tail and bovine dermal collagen type I at four different concentrations were used to assess the impact of 3D scaffold inhomogeneities on cancer cell invasiveness properties. Local inhomogeneities are discontinuities in the structure of the networks. Researchers determined the elastic modulus with AFM in combination with pore size analysis using confocal laser scanning microscopy, which revealed distinct inhomogeneities within collagen matrices. The invasiveness of three breast cancer cell types on these scaffolds varied with different levels of matrix scaffold inhomogeneity, which also influenced the invasion depth that cancer cells achieved. Together, the local matrix scaffold inhomogeneity, pore size, and stiffness of the collagen matrices can affect cell migration [131].

On the other hand, cancer cells directly affect the matrix structure. To investigate this, type-I collagen scaffolds were used as an in vitro 3D biomimetic model to study the effects of MDA-MB-231 or MCF-7 breast cancer cells on matrix. MDA-MB-231, which belongs to the aggressive basal-like subtype, increased the scaffold stiffness and overexpressed the matrix-modifying enzyme lysyl oxidase (LOX), whereas luminal A MCF-7 cells did not significantly alter the mechanical characteristics of extracellular collagen. Mechanical testing was performed using a loading device built in-house to apply a state of unconfined uniaxial compression to the collagen scaffolds. When LOX activity was blocked, the ability of MDA-MB-231 to alter scaffold stiffness was impaired. This replicates the behavior of in vivo orthotopic tumors generated by MDA-MB-231 in female immunodeficient mice, which was characterized by a higher collagen content and higher LOX levels than MCF-7 [132].

Overall matrix mechanics affect cell behavior, but the local mechanical properties of single fibrillar components play a dominant role in regulating cancer cells. By independently controlling fibril diameter and intrafibrillar crosslinking of 3D collagen matrices, researchers showed how fibril bending stiffness instructs cell behavior of invasive and non-invasive breast cancer cells. They reported that changing the fibril thickness or intrafibrillar crosslinking is sufficient to regulate cell behavior over a broad parameter range in terms of morphology, clustering, and invasiveness. For example, higher collagen type-I fibril bending stiffness promoted a more elongated cell shape and higher invasiveness in MDA-MB-231 while decreasing clustering but increasing invasiveness in MCF-7 [133].

To conclude, matrix stiffness cues can be easily tuned in porous scaffold systems, mainly by varying crosslinking types or percentages. The overall mechanics can be analyzed by rheology or tensile/compression testing, while AFM is easily run on the cell surface. These tests need to be further optimized not only for dry but also wet conditions since the final model system includes both the scaffold’s material and cells within liquid media.

## 3. Organoids

Organoid development was derived from the combination of engineering and biology, resulting in a platform with elaborate conformations of cells interacting in a multi-dimensional system inspired by the self-organizing features of an organ during development in vivo [134].

Organoid formation requires embedding and culturing of stem cells or progenitor cells (adult-tissue-resident cells or embryonic progenitors) in a 3D medium. The most-used 3D media are Matrigel and Cultrex BME (a laminin-rich ECM secreted by the Engelbreth-Holm-Swarm tumor line) [135]. Once the cells are encapsulated in the biomaterial, a cocktail of nutrient factors and signals drive the cells to differentiate within the 3D environment, closely resembling the tissue they were derived from [136]. This is considered to be one of the more physiologically relevant 3D culture models because it forms according to intrinsic developmental programs, resulting in remarkable tissue morphology fidelity; it can easily incorporate multiple cell types; and it partially recapitulates organ function [137]. This versatile technology has led to the development of many novel human cancer models, creating the possibility to indefinitely expand organoids starting from the tumor tissue of individuals suffering from a range of carcinomas or using CRISPR-based gene modification to allow the introduction of any combination of cancer gene alterations to normal organoids [138].

Organoids are powerful tools for modeling human organogenesis, homeostasis, injury repair, and disease etiology, but their lack of vasculature, scalability problems, and inherent unpredictability in their self-organization processes represent some of the obstacles they face. Modeling tissue mechanobiology in those systems is fundamental to fully recapitulate tissue features [139].

To address this feature, nanoparticles (NPs) were adopted to engineer the mechanical microenvironment in organoids [140]. They can be dispersed in the matrix with a controlled distribution (local or global) and affect mechanical forces, but they exhibit heterogeneity in magnitude and direction, similar to what occurs in vivo. NPs can also impose force at a cellular level after internalization by target cells. Modulation by NPs of the mechanical stresses imposed on tissues or cells through the control of the external activation field (electric, optical, acoustic, or magnetic) is continuous and reversible. For example, a magnetic field can allow for the directed assembly of gel-dispersed magnetic particles into chains, stiffening the mechanical properties of the microenvironment (modulation of storage modulus between ~0.1 and ~80 kPa reversibly), resulting in mesenchymal stem cells’ increased proangiogenic potential and initiation of osteogenic signaling during early stages of culture on magnetically stiffened substrates [141].

A recent study reported that matrix stiffness has an impact on organoid growth and stem cell signaling in intestinal organoids. The authors reported employing a synthetic scaffold design using a PEG backbone with a consistent and chemically defined synthetic hydrogel to allow reversible modulation of mechanics. Higher stiffness was initially required for YAP activation and intestinal stem cell expansion, and subsequently reduced stiffness, thanks to hydrolytically degradable polymer incorporation, was needed to alleviate the accumulation of compressive forces and permit in vitro organogenesis [142].

In another study, intestinal stem cell colonies were encapsulated into allyl-sulfide hydrogels, and their survival was shown to be stiffness-dependent, indicating mechanosensitivity. Because YAP and Notch signaling are key players in colony formation and crypt maintenance, they utilized controlled photodegradation to facilitate intestinal organoid differentiation, showing that the size and number of intestinal crypts were dependent on the extent of matrix softening [143].

As discussed, organoids recapitulate many structural and functional aspects of their in vivo counterparts. This technology is unique, based on the model’s self-organizing properties, and high similarity to—and in some cases, histologically indistinguishable from—actual human organs [144]. Furthermore, if combined with immune cells and fibroblasts, tumor organoids can model the cancer microenvironment, enabling immune-oncology applications. In this exciting and evolving field, organoids can be also used to accurately predict drug responses in a personalized treatment setting [138].

## 4. Microfluidic Organs on Chips

In recent years, cutting-edge technologies have allowed researchers to break down the micro-scale barrier. Microfluidic platforms seem to be one of the most promising tools in this area [145]. Nanostructured devices can be designed with unprecedented precision and give researchers the possibility to replicate different organs and tissue features. Among those, organs on chips (OoCs) represent promising in vitro models to combine crucial dynamic mechanical cues as well as chemical signals for complex 3D organ-like structure reproduction [146].

Several different designs have been developed so far, but some specific characteristics are present in each OoC system. Each OoC is composed of a polymeric structure, usually flexible and transparent, specifically designed to encase hollow microfluidic channels lined by living cells. The most common material used for developing such a system is polydimethylsiloxane (PDMS). PDMS is biocompatible, cheap, and transparent, and has been used in several biomedical applications. In addition, its gas permeability paired with the possibility to mold it with a resolution of a few nanometers have expanded its use to microfluidic applications.

OoC platforms have aimed to combine cancer cells, stromal cells, and vasculature to mimic TME complexity in human pathological and physiological conditions [147,148]. For example, Liu et al. built a microfluidic device to study the interactions between salivary gland adenoid cystic carcinoma (ACC) cells and cancer-associated fibroblasts (CAFs), a stromal-activated cell type usually associated with tumor cells. The device contains six co-culture units connected by a medium channel. The two cell types were seeded in two separate compartments to allow communication only through the medium diffused in the matrix. The authors were able to replicate the in vivo invasion pattern of ACCs regulated by CAFs and quantify and measure the distance and area covered by cancer cells during invasion in a spheroid-like fashion [149]. One of the limitations of this approach is the lack of vasculature, which is crucial to mimic cancer cell migration through different barriers in vivo. Further complexity can be achieved in those systems; for instance, mechanobiology-on-a-chip is drawing attention in cancer research because it focuses on how mechanical inputs modulate biological and chemical outputs in microphysiological systems [150].

Several strategies have been exploited throughout the years to add biomechanical cues to OoC systems, ranging from testing specific ECM types with tunable characteristics (e.g., biocompatibility, stiffness, porosity, and degradation rate) to the application of shear stresses and active stretch/strain or compression forces using integrated flexible membranes. These systems not only reproduce tissue biophysical forces but also allow modulation of the intensity and duration of such forces. Marsano et al. pioneered the design of a 3D microfluidic system, the so-called beating heart-on-chip, for the generation of mature and highly functional micro-engineered cardiac tissues. The device is composed of an array of hanging posts, specifically designed to confine cell-laden gels and allow stretching of the cell constructs when stimulated, and a pneumatic actuation system to induce homogeneous uniaxial cyclic strain to the 3D cell constructs during culture. The cell construct, constituted by human induced pluripotent stem cell-derived cardiomyocytes (hiPSC CM) embedded within an artificial ECM made of fibrin gel, was subjected to mechanical stimulation by cyclic strain following a specific pattern (10% uniaxial strain, 1-Hz frequency). The mechanical stimulation was achieved using inflatable micro-membranes located under the cell construct, exerting a micrometric displacement. Interestingly, they were able to observe a cardiac-like morphology in the stimulated cells and the expression of specific cardiac markers, suggesting successful conditioning. This system demonstrates how biochemical cues can generate phenotypic changes and promote cell differentiation [151]. However, the size of the off-chip actuation system could impact sterility when used for long culturing periods.

The lack of non-invasive sensors to precisely evaluate in situ the key biomechanical cues applied, as well as the mechanical properties of the devices developed, is a common limitation among the OoC models designed so far. Since the ECM is of paramount importance in the differentiation and proliferation of normal cells as well as in the progression of diseases such as cancer, sensing systems able to measure ECM properties are needed. Zareei et al. recently developed an ultrasonic platform that can assess ECM stiffness in real time in a non-destructive fashion. The device is composed of a pair of millimeter-scale ultrasonic transmitter and receiver transducers with the test medium placed between them. The functional principle relies on the generation of an ultrasonic wave that passes through the targeted material (e.g., ECM). On the other end, a piezoelectric receiver detects the wave and generates a corresponding electric signal that carries the information regarding the stiffness of the material. Such a system allows in situ stiffness assessment, evaluating potential variations that can occur in the ECM in long-term culture applications. The authors compared the robustness of the novel measuring systems with standard compression tests, and the results showed an accuracy greater than 93% [152]. However, as underlined by the authors, this platform suffers from some limitations, mainly related to the use of an ultrasonic wave as the probe. Indeed, air bubbles entrapped in the ECM or heterogeneous compounds impact the final measurements, causing distinct errors.

Although the first OoC was developed during the last decade, these platforms are considered “the future” of 3D culturing systems because their micrometric dimensions allow for accurate control of the external and internal cell environment [153]. Furthermore, they allow researchers to exploit diffusion phenomena and biochemical cues generated by the cells themselves, reducing the gap between the model and in vivo conditions. Traditional in vitro models do not recapitulate an environment in which cells are surrounded by a multitude of stimuli and each cue carries information. OoCs have been included in the list of the top 10 emerging technologies in the World Economic Forum, but the extensive expertise required to use them for a multidisciplinary approach is slowing down their widespread usage in research [154]. The current necessity for high-throughput systems in drug screening and drug development in the pharmaceutical industry, together with increasing ethical concerns regarding the use of animal models, have accelerated the transition from 2D systems to 3D in vivo-like models, like OoC [41]. On-chip sensing systems, the implementation of vasculature within the chips, and the recapitulation of biomechanical cues are just some of the open challenges that need to be addressed by OoC devices, but the promising results obtained so far are leading to the establishment of a new standard in the life science field. Several research groups have started to investigate how mechanical cues affect gene expression and how such inputs lead to phenotypic changes, but there is still room for improvement, and numerous questions need to be addressed. A better understanding of all these underlying details, coupled with epigenetic studies, could pave the way for breakthrough findings in cancer research.

## 5. Conclusions

Tumor development is a dynamic process orchestrated by cellular crosstalk and interaction with the surrounding matrix in a 3D context. The complexity of these mechanisms relies both on the number of players exchanging signals and the different nature of those signals (biochemical, biological, and biophysical), which influence cancer cell fate. Understanding the key underlying changes happening during tumor initiation and progression is necessary to develop efficient diagnostic methods and treatments. To achieve this goal, we need to deconstruct complexity into simpler and more predictable systems. Widely used as in vitro models, 2D culture systems fail at reproducing physiological conditions, leading to experimental inconsistency, lack of reproducibility, and a very poor level of complexity. To avoid inconclusive and misleading results, a third dimension was included in in vitro models, sometimes coupled with the use of additional substrate material, which may or may not be biologically active, as structural support for cell growth.

The first steps have been focused on the generation of multicellular spheroids that allow the formation of a core with hypoxic and quiescent cells. Growing as independent cellular aggregates, they mimic anticancer drug resistance compared to conventional cultures [155] but fail to reproduce cancer–environment interactions. In this framework, ECM components have been introduced to expose cells to appropriate physical, chemical, and mechanical cues. These cultures have reported significant phenotypic and behavioral differences between normal and metastatic epithelial cells.

Different materials are exploited to model different tumor stages. Collagen, Matrigel, and hyaluronic acid materials have been the most common natural materials used for modeling and studying both primary and invasive tumors; as platforms to mimic either the processes like ECM degradation, migration, and the epithelial–mesenchymal transition; and the advanced process of intravasation, extravasation, and metastasis through the mesenchymal–epithelial transition (MET). Synthetic polymers, such as PEG or nanofiber scaffolds (RAD16-I) functionalized with adhesive/recognition sites for integrin binding or protease degradation, are also useful to study the effect of tumors on ECM [156]. Perhaps the most exciting developments have been the recent OoC methods, which allow for the construction of connected chambers that mimic different organ compartments, for example, liver ducts and blood vasculature [157]. Tumor-on-chip platforms have been designed to recreate controllable culture environments, mainly to investigate the blood circulation, drug delivery, intravasation, and extravasation processes occurring during tumor progression. Unfortunately, the extensive user training required for multistep fabrication, specific set-up equipment, small-volume culture and staining protocols, and difficulties in recovering seeded cells for further characterization represent a few of the disadvantages of using such platforms.

Overall, 3D cultures could represent a highly informative and effective model to optimize drug candidates, mimicking native tissue distribution, and reduce animal testing, improving cost-effectiveness and avoiding ethical concerns. To this aim, 3D models need to gain high-throughput applicability, simple and standardized culture protocols and analysis techniques, and high-resolution imaging (Figure 2).

Indeed, many microscopy techniques can only be applied to imaging transparent matrix gels. More advanced analysis approaches need to be developed and applied for non-transparent scaffolds. Furthermore, 3D microenvironments lack vasculature and hence both nutrient supply and the normal transport of small molecules; unlike continuous in vivo models, they mimic static or short-term conditions and do not model interactions with other cell types or the influence of the latter upon the culture. Related to this, and fundamental to mimic a living tissue and a more natural ECM secretion, is the inclusion of cell types such as fibroblasts, endothelial cells, and mesenchymal stem cells in coculture with cancer cells to enable the production of endogenous ECM by the stromal cells. Furthermore, the inclusion of primary patient-derived cells will enable the development of more accurate 3D models, retaining the patient and tumor characteristics and more appropriately reflecting tumor heterogeneity in the population [158]. Interestingly, biobanks of patient-derived 3D cancer models could refine our understanding of interpatient as well as intrapatient heterogeneity, paving the way for personalized cancer therapies.

Finally, matrix chemical composition and physical properties should be optimized with reference to natural tissue properties. Three-dimensional system design should progress to highly resemble the tissue being studied in order to fill the gap between in vitro and in vivo models. A good 3D platform will evaluate the efficacy of anticancer agents, discover potential target genes for therapy, and reveal signaling pathways relevant for tumor progression.

We explored different 3D culture techniques in this review, showing their potential in the study of mechanosensing, although the selection of one model over another is highly contextual and depends on the studied biological questions (Figure 2).

## 6. Future Directions

Cancer models span from uni-/multicellular spheroids, cancer-on-a-chip, organotypic slices of cancer tissues, and hydrogel- and scaffold-based systems to self-assembly (no exogenous scaffold needed) techniques (Figure 2). Although many questions and hurdles remain about 3D models’ accessibility, reproducibility, and integrity, increasing the complexity of the TME represents one of the next important challenges in the field. The multicellularity required to model this environment necessitates the incorporation of stromal cells and immune cells together with a vascular/lymphatic network simulating dynamic blood flow and providing mechanical signals regulating tumor development and function.

The future of medicine appears to be precision and personalized medicine. Precision medicine approaches patient care on the basis of a genetic understanding of their disease (e.g., blood transfusion according to blood typing and autologous grafting), targeting specific disease variants. Personalized medicine focuses on providing patient-tailored therapies. In this framework, patient-based 3D models display unique features as a result of the cell donor’s genetic and epigenetic backgrounds, lifestyle, and medical history, so the models can be used to evaluate drug efficacy and responses as a precision medicine approach.

The inclusion of 3D model systems in bioreactors will provide close control and monitoring of the environment (e.g., temperature, pH, nutrient supply, and waste removal), together with higher reproducibility and automation. Media flow systems will allow for circulation of nutrients, removal of wastes, and homogeneity of the environment within the reactor, ideal for high-volume cell production and ex vivo tissue engineering applications. Alongside bioreactors, progresses in 3D bio-printing will improve the diversity, fidelity, and capacity of 3D culture models in cancer research. Three-dimensional bio-printing techniques can generate geometric constructs containing viable cells but can also simplify high-throughput applications with precise reproducibility [159].

Sometimes, the scaffold itself can be an innovative therapeutic strategy. The concept of creating an artificial niche can be integrated with the potential to capture tumor cells actively disseminating in the peritoneal cavity to create a therapeutic strategy modulating the interactions of metastatic cells with the ECM. This idea was tested with the aim of transforming a disseminated disease into a focal disease. Researchers developed a “biomimetic” ECM composed of a non-resorbable 3D scaffold with collagen coating on different murine preclinical models of advanced ovarian cancer, showing the possibility to control peritoneal carcinomatosis upon primary ovarian debulking surgery and to expand the percentage of patients who are candidates for second rescue surgeries at the time of relapse [160].

Furthermore, investigations into these systems will provide new insight into the tumor matrix, creating a common ground to tackle solid cancers, reconfiguring the cancer matrisome while identifying new matrix-related targets for drug delivery to the tumor site. Finally, 3D culture systems paired with patient-derived xenografts or patient-derived organoids could represent a clinically relevant platform toward truly personalized research, therapies, and drug development for cancer patients [161].

Overall, these platforms represent a novel, reliable preclinical patient-specific platform to bridge the gap between in vitro and in vivo drug testing assays, providing preclinical evaluation of drug cytotoxicity, efficacy, and efficiency for effective cancer treatment [162,163,164,165,166,167,168,169]. In nanomedicine, these models can be used to study nanoparticle drug delivery, mechanical modulation and imaging, ECM–nanoparticle interactions, nanoparticle diffusion in the ECM, and which cell types internalize the nanoparticles [170,171,172].

Not only cancer research but also fields such as regenerative medicine will benefit from the use of different 3D biomaterials to effectively support cell culture, improve cell transplantation, and as platforms for drug research on drug screening [173].

Three-dimensional cell culture approaches hold great promise for various purposes, ranging from disease modeling to drug discovery and cancer-targeted therapy. With all the advantages of 3D monoculture and coculture systems, the insights they provide will increase our understanding of the tumor micro-milieu while developing and testing new cancer therapies in vitro, attacking two possible targets: the tumor cell and its environment.

## Abbreviations

ECM	Extracellular matrix
TME	Tumor microenvironment
LINC	Linkers of nucleoskeleton and cytoskeleton proteins
NPCs	Nuclear pore complexes
LBR	Lamin B receptor
AFM	Atomic force microscopy
ROCK	Rho-associated protein kinase
FAK	Focal-adhesion kinase
PEG	Polyethylene glycol
rBM	Reconstituted basal membrane
WDR5	WD repeat-containing protein 5
BME	BM extract
TSA	Trichostatin A
CAFs	Cancer-associated fibroblasts
TSPBA	N1-(4-boronobenzyl)-N3-(4-boronophenyl)-N1,N1,N3,N3-tetramethylpropane-1,3-diaminium
µTAMs	Micro scale temperature-actuated mechanosensors
PCL	poly(ε-caprolactone
PGA	Poly(glycolic acid)
PLA	Poly(lactic acid)
LOX	Lysyl oxidase
NPs	Nanoparticles
OoCs	Organs on chips
PDMS	Polydimethylsiloxane
hiPSC CM	Induced pluripotent stem cell-derived cardiomyocytes
MET	Mesenchymal–epithelial transition
